# Computational metabolomics – a field at the boundaries of cheminformatics and bioinformatics

**DOI:** 10.1186/1758-2946-3-S1-O6

**Published:** 2011-04-19

**Authors:** Christoph Steinbeck, Stefan Kuhn, Kalai Vanii Jayaseelan, Pablo Moreno

**Affiliations:** 1 Cheminformatics and Metabolism Group, European Bioinformatics Institute (EBI), Cambridge, CB10 1SD, UK

## 

Metabolomics studies the occurrence and change of concentrations of small molecular weight chemical compounds (metabolites) in organisms, organs, tissues, cells and ultimately cell compartments in the context of environmental changes, disease or other boundary conditions. It does this by means of spectroscopic and chromatographic techniques and by observing at once not only a few but all compounds visible to the particular technique used. As such, it is not only the third large Omics area of research next to Genomics and Proteomics, but also a field at the boundary between chemistry and biology, helping to answer biological questions using analytical chemistry and cheminformatics techniques.

This talk gives an overview about the field of Metabolomics and the challenges we face with respect to computing, informatics as well as chemical information aspects of metabolomics, including the analysis of metabolomics experiments, metabolomics databases, computer-assisted structure elucidation of metabolites and more. We give examples from our own research where we have for instance used machine learning techniques to predict NMR spectra of biological metabolites and applied those in computer-assisted structure elucidation systems for metabolites [1-3] – a classical area of cheminformatics which is now seeing a renaissance in the context of metabolomics. We further outline the data-collection challenges currently being address by the development of the MetaboLights database in our group at the European Bioinformatics Institute.
